# High cholesterol absorption efficiency interferes with bile acid metabolism and cholesterol elimination from the body

**DOI:** 10.1111/joim.70031

**Published:** 2025-10-20

**Authors:** Piia Simonen, Ingmar Wester, Jyri Lommi, Juha Sinisalo, Helena Gylling

**Affiliations:** ^1^ Heart and Lung Center, Cardiology Helsinki University Hospital and University of Helsinki Helsinki Finland; ^2^ Raisio Group plc Raisio Finland

**Keywords:** atherosclerosis, bile acids, cholesterol absorption, cholesterol synthesis, low‐density lipoprotein cholesterol, reverse cholesterol transport

## Abstract

**Background:**

Elevated low‐density lipoprotein (LDL) cholesterol causes atherosclerotic cardiovascular diseases. Variables of whole‐body cholesterol metabolism, for example, high cholesterol absorption efficiency, might also be atherogenic, whereas the role of bile acids is controversial.

**Objectives:**

This post hoc study concerns the impact of cholesterol absorption on bile acid metabolism. The hypothesis was that cholesterol absorption efficiency interferes with bile acid metabolism.

**Methods:**

Cholesterol metabolism was studied using absolute and relative methods. Elimination of cholesterol from the body as bile acids and neutral sterols was assessed from 24‐h faecal collections and analysed by gas–liquid chromatography. Cholesterol absorption efficiency was evaluated by a peroral continuous dual‐isotope feeding method, and cholesterol synthesis by a sterol‐balance technique. The relative methods included analyses of serum biomarkers of cholesterol absorption efficiency and cholesterol synthesis by gas–liquid chromatography.

**Results:**

Faecal bile acids, neutral sterols and cholesterol synthesis were lower in high‐ versus low‐cholesterol absorbers. Elimination of cholesterol from the body as bile acids and neutral sterols was reduced in high‐ versus low‐cholesterol absorbers. Serum and LDL cholesterol levels did not differ in low‐ versus high‐cholesterol absorbers. Absolute and relative methods of cholesterol metabolism correlated with each other, suggesting that the results can be considered valid.

**Conclusion:**

In high‐cholesterol absorbers, poor elimination of cholesterol from the body as bile acids and neutral sterols may indicate an increased risk of atherosclerosis. It can be prevented by decreasing cholesterol absorption and increasing reverse cholesterol transport by dietary means combined with ezetimibe and statin treatment, when needed.

## Graphical abstract

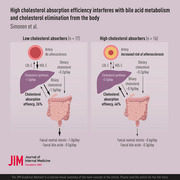

**Abbreviations**
LDL‐C: low‐density lipoprotein cholesterol
HDL‐C: high‐density lipoprotein cholesterol

## Introduction

Atherosclerotic cardiovascular diseases (ASCVDs), especially coronary artery disease (CAD), are common causes of morbidity and mortality globally [1]. They are mainly caused by elevated levels of plasma apoprotein B 100 (apoB100)‐containing low‐density lipoproteins (LDLs) [2], but also, for example, lipoprotein (a), and some variables of whole‐body cholesterol metabolism are suggested to have atherogenic potential [2, 3, 4, 5, 6, 7, 8, 9, 10, 11]. Cholesterol metabolism, including cholesterol absorption, cholesterol and bile acid synthesis, and cholesterol elimination from the body via bile as faecal neutral sterols of cholesterol origin and as bile acids, transports large influx‐ and efflux‐volumes of cholesterol in the body under tight homeostatic regulation [12, 13, 14, 15].

The atherogenicity of cholesterol metabolism was evaluated in 86 nondiabetic individuals by correlating the variables of cholesterol metabolism with carotid intima‐media thickness (CIMT) [8]. Cholesterol absorption efficiency was associated positively with CIMT (*r* = 0.279, *p* = 0.0092), whereas cholesterol synthesis (*r* = −0.425, *p* < 0.0001) and biliary cholesterol elimination as faecal neutral sterols were associated negatively with it (*r* = −0.472, *p* < 0.0001), suggesting that high cholesterol absorption efficiency was atherogenic, but high cholesterol synthesis and high faecal neutral sterol elimination were atheroprotective [8]. Similar results have also been documented in other studies [4, 5, 6, 7, 9, 10, 15, 16]. However, faecal elimination of bile acids has a controversial role in the increased risk of atherosclerosis [8, 17, 18].

Furthermore, in two studies, including separately only women or men, it turned out that the presence of CAD, but not, for example, gender, body mass index (BMI), serum and lipoprotein lipids or blood glucose concentration, modified the profile of cholesterol metabolism [4, 5]. Accordingly, postmenopausal women with stable CAD had lower cholesterol synthesis and lower biliary cholesterol elimination as neutral sterols compared with their age‐matched controls without CAD (*p* < 0.05) [4]. Men with CAD and Type 2 diabetes had higher cholesterol absorption efficiency compared with their age‐matched controls without CAD (*p* < 0.05) [5]. Cholesterol absorption efficiency and serum biomarkers of cholesterol absorption were the only variables significantly associated with CAD [5]. Thus, the presence of CAD independently altered cholesterol metabolism in women and men.

Cholesterol absorption efficiency and faecal neutral sterol elimination are genetically regulated and coordinated mainly via liver X nuclear receptors [19, 20, 21], suggesting that cholesterol absorption efficiency and faecal neutral sterol elimination have important roles in the potential atherogenicity and anti‐atherogenicity in whole‐body cholesterol metabolism. Cholesterol absorption efficiency is considered to be high when it varies between 50% and 60% or more, and the prevalence of high absorbers is estimated to be ∼30% in different populations [22, 23]. High cholesterol absorption efficiency is caused by loss‐of‐function genetic variations in the small intestine ATP‐binding cassette transporters ABCG5 and ABCG8 [9, 11], whereas loss‐of‐function genetic variations in the hepatic ABCG5 and ABCG8 transporters diminish hepatobiliary cholesterol efflux. Interestingly, if the genetic variations in the small intestine and hepatic ABCG5 and ABCG8 transporters decrease the circulating LDL cholesterol (LDL‐C) concentrations, the cumulative incidence of myocardial infarction is decreased, but that of gallstone disease is increased [24].

Bile acids are sterols synthesized from cholesterol in hepatocytes and excreted to bile, but 95% of them are reabsorbed in the terminal ileum [21]. Thus, the daily faecal elimination of bile acids reflects their 24‐h synthesis. Their synthesis provides the primary means to catabolize cholesterol, but they also facilitate the absorption of lipids and participate as regulators in lipid and glucose metabolism [21]. The first and rate‐limiting step in the classic pathway of bile acid synthesis is the conversion of cholesterol into 7α‐hydroxycholesterol by the enzyme cholesterol 7α‐hydroxylase (CYP7A1) [21]. It is regulated by the negative feedback of bile acid reabsorption, so that interruption in the enterohepatic circulation by drugs or by dietary means increases the activity of CYP7A1. Also, hydroxymethylglutaryl coenzyme A reductase (HMG CoA‐R), the rate‐limiting enzyme of cholesterol synthesis, and CYP7A1 are closely coupled so that an increase in cholesterol synthesis is paralleled with the degradation of cholesterol into bile acids [25]. CYP7A1 is coordinated via the liver farnesoid X receptor [21, 26].

The purpose of this post hoc study, combining the results of two earlier original trials [27, 28] (Table S1), was to investigate the impact of cholesterol absorption efficiency, in particular on bile acid metabolism. The study population (*n* = 33) was divided into low‐ and high‐cholesterol absorbers, and the metabolism of cholesterol and bile acids was analysed in these two subgroups. Unfortunately, only 21 blood samples were available from the CAD women for the analyses of the present study, but in men with Type 2 diabetes, detailed characteristics and blood samples were available from 12 men. Thus, the present study population contained 12 men with Type 2 diabetes and 21 women with stable CAD (Table 1). The hypothesis was that different profiles of cholesterol absorption efficiency, that is, low‐ versus high‐cholesterol absorption efficiency, interfere with bile acid synthesis.

**Table 1:** Characteristics of the study population, dietary cholesterol and fat intake and serum and lipoprotein lipids in the whole study population and after division into low‐ and high‐cholesterol absorber subgroups.

Variables	All *n* = 33	Low‐cholesterol absorbers *n* = 17	High‐cholesterol absorbers *n* = 16	*p‐*values
Men/Women, *n* (%)	12/21 (36/64)	11/6 (65/35)	1/15 (6/94)	<0.001
Age, years	55.2 ± 4.67	56.5 ± 4.74	53.8 ± 4.19	0.108
Body mass index, kg/m^2^	26.1 ± 3.27	26.5 ± 2.35	25.6 ± 3.92	0.465
Dietary cholesterol, mg/day	239 ± 107	262 ± 125	215 ± 73.6	0.212
Dietary fat, g/day	75.5 ± 22.7	80.8 ± 26.9	67.2 ± 12.9	0.083
Serum and lipoprotein lipids, mmol/L				
Serum cholesterol	6.04 ± 0.93	5.90 ± 0.72	6.07 ± 0.89	0.568
LDL cholesterol	3.72 ± 0.75	3.64 ± 0.52	3.81 ± 0.90	0.523
HDL cholesterol	1.22 ± 0.25	1.20 ± 0.24	1.24 ± 0.25	0.639
Serum triglycerides	1.64 ± 0.74	1.88 ± 0.58	1.38 ± 0.77	0.052

*Note*: Mean ± standard deviation. The participants were divided into low‐ and high‐cholesterol absorbers on the basis of median cholesterol absorption efficiency of the whole study population during the baseline rapeseed‐oil control period [27, 28]. The median cholesterol absorption efficiency in the low cholesterol absorbers was ≤34.5%, range 11.9%–34.5%, and in the high cholesterol absorbers >34.5%, range 35.1%–63.5%.

Abbreviations: HDL, high‐density lipoprotein; LDL, low‐density lipoprotein.

## Methods

### Study population

The original study populations were recruited from outpatient clinics of the Department of Medicine, University of Helsinki [27, 28] (Table S1). The inclusion criteria included individuals with mild to moderate hypercholesterolaemia without cholesterol‐lowering medication, and the individuals were symptomless and in good and stable overall health. All participants volunteered to take part in the study.

The complicated and laborious methods and analyses of the absolute measurements of whole‐body cholesterol metabolism restrict in general the size of the study population. To keep the size limited, we selected the study populations with as wide and different distributions as possible of cholesterol absorption efficiency and cholesterol synthesis. The first original study included only men with Type 2 diabetes under good glycaemic control and without CAD (*n* = 11) [27], and the second study included only women without Type 2 diabetes but with stable CAD (*n* = 22) [28] (Table S1). Age, BMI, serum total, LDL‐ and HDL‐C concentrations were similar between the groups, but very low‐density lipoprotein (VLDL)‐C and serum triglyceride concentrations were higher in men versus women. Cholesterol absorption efficiency was higher, but cholesterol synthesis, faecal neutral sterol elimination and faecal bile acids were significantly lower in women with CAD versus men without CAD. Also, according to earlier studies [4, 5], the presence of CAD independently altered cholesterol metabolism in women and men, suggesting that the differences in gender between the study populations did not interfere with the outcomes of cholesterol metabolism.

### Study design

The original studies were double‐blind, randomized, controlled clinical trials using rapeseed oil margarine without (control period) and with phytostanol esters (phytostanol ester period). Detailed descriptions of the study procedures are available in the corresponding original publications [27, 28]. In this post hoc study, only the results from the control periods of the original crossover studies were included. In brief, after the run‐in period of ad libitum home diet, the participants replaced 30 and 21 g of their daily total fat intake with rapeseed oil‐based margarine for 6 [27] and 7 weeks [28].

### Outcome measurements

Blood samples were drawn after a 12‐h fast at the end of the control period [27, 28]. Metabolic studies included assessments of cholesterol absorption efficiency, cholesterol synthesis and biliary elimination of endogenous cholesterol as faecal neutral sterols and faecal bile acids. The faecal neutral sterols also included the cholesterol‐derived compounds coprostanol and coprostanone. Faecal bile acids denote their de novo synthesis [21].

The participants were given capsules containing ^14^C‐cholesterol, ^3^H‐sitosterol and chromic oxide (Cr_2_O_3_) for the last 7 days of the control period in order to assay cholesterol absorption efficiency and the amount of faecal flow. At the same time, they kept 7‐day food diaries and carried out 3‐day faecal collections.

Serum concentrations of total cholesterol, noncholesterol sterols, including the cholesterol precursors zymostenol and lathosterol, cholestanol (a metabolite of cholesterol) and sitosterol (a phytosterol), were assayed by gas–liquid chromatography (GLC) (Ultra 2, Agilent Technologies) and flame ionization detection with 5α‐cholestane as an internal standard [29]. Serum concentrations of the noncholesterol sterols were adjusted to those of serum total cholesterol analysed in the same GLC run and expressed as ratios to cholesterol (10^2^ µmol/mmol of cholesterol). The noncholesterol sterols are called ratios to cholesterol herein (for example, lathosterol:C). As ratios to cholesterol, they are validated serum biomarkers of cholesterol synthesis and cholesterol absorption efficiency [30, 31, 32, 33, 34]. Serum and lipoprotein lipids were assayed at the Diagnostic Center, Helsinki University Hospital Laboratories, using the accredited photometric and direct enzymatic methods. Unfortunately, detailed information of the methods is not available.

Cholesterol absorption efficiency was evaluated by a peroral continuous dual‐isotope feeding method, in which the altered ^14^C/^3^H ratio in faeces was compared with the ratio in the ingested capsules [35]. Quantification of Cr_2_O_3_ indicated the amount of faecal flow [36]. Dietary intakes of cholesterol and fatty acids were analysed by means of a computerized method using data from 7‐day food diaries [37].

Cholesterol synthesis was analysed using the sterol‐balance method [38]. The method is based on the prerequisite of a metabolic steady state, denoting that cholesterol synthesis is equal to the difference between the intake of cholesterol and the faecal elimination of cholesterol‐derived compounds. Faecal neutral sterols and bile acids were analysed by GLC [39, 40]. Of the individual bile acids, cholic and chenodeoxycholic acids (the primary bile acids) and deoxycholic and lithocholic acids (the secondary bile acids) were analysed, and the rest of the total bile acids were combined as miscellaneous bile acids.

### Statistical analyses

Statistical analyses were performed by using IBM SPSS for Windows 22.0 software (IBM SPSS, Chicago, IL). Sample‐size calculation was based on significance levels (*α* = 0.05 and *β* = 0.20). Using these estimates, the preset 80% statistical power was achieved, and the size of the required population was appropriate. Normality and homogeneity of variance assumptions were checked before further analyses, and variables not normally distributed were transformed logarithmically. Continuous variables were tested by using Student's *t*‐test and the paired *t*‐test. Spearman's rank correlation coefficients were calculated. A two‐sided *p*‐value of <0.05 was considered statistically significant. The results are expressed as mean ± standard deviation.

## Results

### Study population

The whole study population of the present study included 33 individuals, of whom 12 (36%) were men with Type 2 diabetes and 21 (64%) were women with stable CAD (*p* < 0.001) (Table 1).

In the whole study population, the mean age was 55.2 years (range 49–67 years), and the mean BMI was 26.1 kg/m^2^ (range 20.9–33.0 kg/m^2^). The mean dietary cholesterol intake was 239 mg/day (range 114–607 mg/day), and that of dietary fat was 75.5 g/day (range 44.0–146 g/day). The mean serum cholesterol concentration was 6.04 mmol/L (range 4.46–7.82 mmol/L), and that of LDL‐C was 3.72 mmol/L (range 2.51–5.73 mmol/L).

**Fig. 1:**
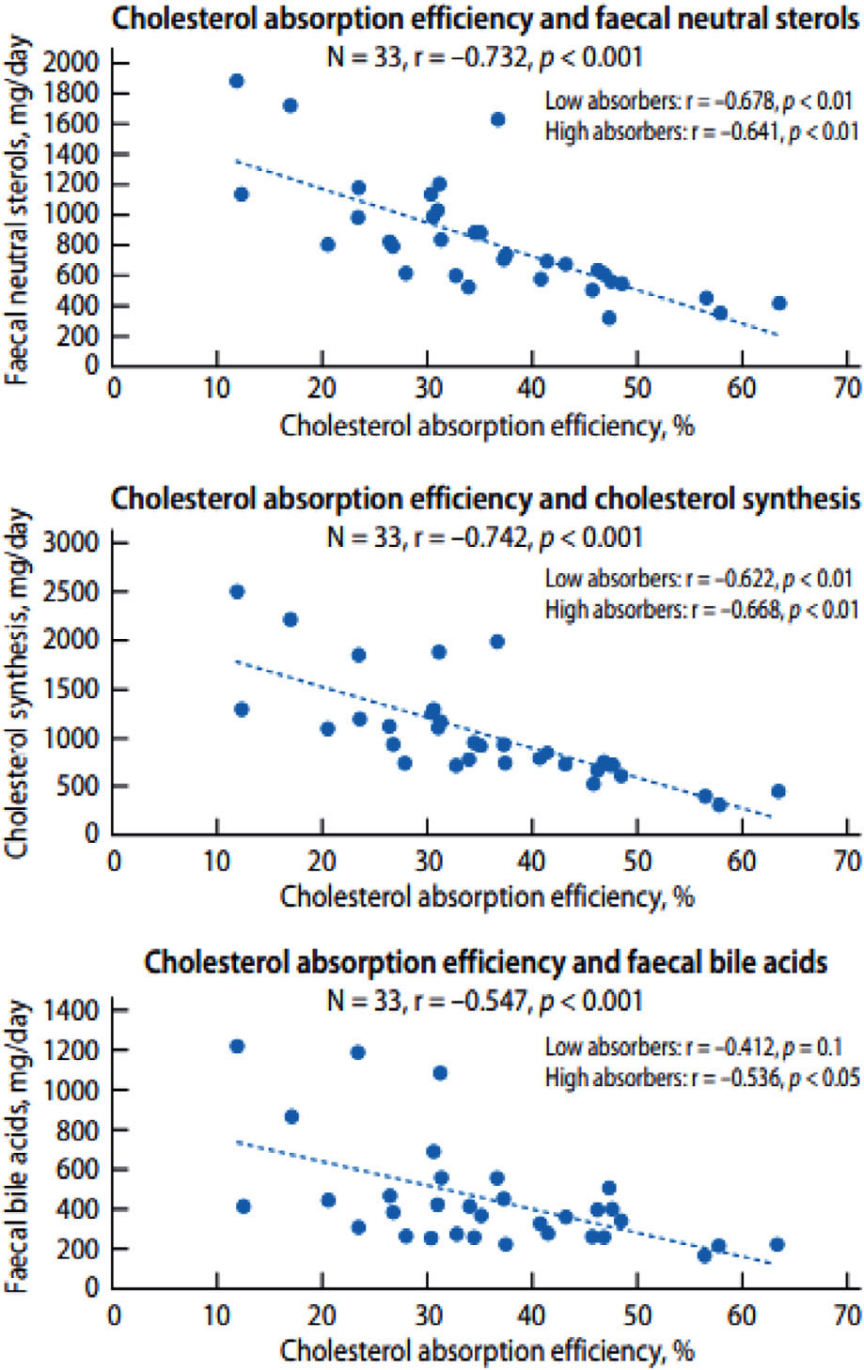
The relationships between cholesterol absorption efficiency and faecal neutral sterols (upper panel), between cholesterol absorption efficiency and cholesterol synthesis (middle panels) and between cholesterol absorption efficiency and faecal bile acids (lower panel).

The whole study population was divided into low (*n* = 17) and high (*n* = 16) cholesterol absorption subgroups based on the median cholesterol absorption efficiency of the whole study population evaluated during the last 7 days of the control period (Tables 1, 2, 3). In the low cholesterol absorbers, the median cholesterol absorption efficiency was ≤34.5% (range 11.9%–34.5%), and in the high absorbers, it was >34.5% (range 35.1%–63.5%) (Fig. 1). Most of the low cholesterol absorbers (65%) were men with Type 2 diabetes, whereas most of the high cholesterol absorbers (94%) were women with CAD (Table 1).

**Table 2:** Serum biomarkers of cholesterol synthesis and cholesterol absorption efficiency and variables of cholesterol and bile acid metabolism in the whole study population and after division into low‐ and high‐cholesterol absorber subgroups.

Variables	All *n* = 33	Low absorbers *n* = 17	High absorbers *n* = 16	*p‐*values
Serum biomarkers of cholesterol synthesis^a^				
Zymostenol:C	20.5 ± 10.7	25.4 ± 11.0	15.3 ± 6.97	0.005
Lathosterol:C	183 ± 57.3	196 ± 57.9	169 ± 51.0	0.168
Serum biomarkers of cholesterol absorption efficiency^a^				
Cholestanol:C	115 ± 38.7	99.4 ± 20.8	131 ± 44.8	0.020
Sitosterol:C	136 ± 53.0	113 ± 35.1	161 ± 56.0	0.009
Variables of cholesterol metabolism				
Cholesterol absorption, %	35.7 ± 12.4	26.2 ± 6.92	45.8 ± 7.82	<0.001
Cholesterol synthesis, mg/day	1039 ± 527	1304 ± 502	756 ± 364	0.002
Faecal neutral sterols, mg/day	831 ± 381	1009 ± 354	641 ± 296	0.004
Variables of bile acid metabolism				
Faecal bile acids, mg/day	447 ± 274	557 ± 324	330 ± 108	0.015
Cholic acid, mg/day	10.9 ± 14.7	16.0 ± 18.8	5.44 ± 3.36	0.042
Chenodeoxycholic acid, mg/day	8.95 ± 6.91	12.5 ± 7.80	5.15 ± 2.42	0.002
Deoxycholic acid, mg/day	161 ± 114	210 ± 138	109 ± 38.7	0.011
Lithocholic acid, mg/day	76.4 ± 53.4	95.5 ± 65.0	56.2 ± 24.7	0.035
Primary bile acids, mg/day	19.8 ± 21.0^b^	28.5 ± 16.0^b^	10.6 ± 5.37^b^	0.015
Secondary bile acids, mg/day	227 ± 163	306 ± 196	165 ± 60.3	0.013
Miscellaneous bile acids, mg/day	190 ± 109	223 ± 132	154 ± 59.9	0.075
Total biliary cholesterol elimination				
Faecal neutral sterols + bile acids, mg/day	1278 ± 597	1566 ± 611	971 ± 366	0.003

*Note*: Mean ± standard deviation. The participants were divided into low‐ and high‐cholesterol absorbers on the basis of median cholesterol absorption efficiency of the whole study population during the baseline rapeseed‐oil control period [27, 28]. The median cholesterol absorption efficiency in the low cholesterol absorbers was ≤34.5%, range 11.9%–34.5%, and in the high cholesterol absorbers >34.5%, range 35.1%–63.5%. Primary bile acids: cholic acid, chenodeoxycholic acid. Secondary bile acids: deoxycholic acid, lithocholic acid. Miscellaneous bile acids: total bile acids minus (primary plus secondary bile acids).

Abbreviation: C, cholesterol.

^a^ 10^2^ µmol/mmol of cholesterol.

^b^ Significantly different from the secondary and miscellaneous bile acids (*p* < 0.0001).

**Table 3:** Correlations between LDL cholesterol concentrations and the variables of absolute metabolism of cholesterol and bile acids in the whole study population and after division into low‐ and high‐cholesterol absorber subgroups.

Variables	All *n* = 33	Low absorbers *n* = 17	High absorbers *n* = 16	*p‐*value
LDL cholesterol, mmol/L	3.72 ± 0.75	3.64 ± 0.52	3.81 ± 0.90	0.523
Correlation coefficients				
Cholesterol absorption, %	0.112	−0.230	0.174	All NS
Cholesterol synthesis, mg/day	−0.127	−0.129	−0.045	
Faecal neutral sterols, mg/day	−0.064	−0.092	0.052	
Faecal bile acids, mg/day	−0.211	−0.246	−0.224	
Faecal neutral sterols + bile acids, mg/day	−0.138	−0.183	−0.024	

*Note*: Mean ± standard deviation.

Abbreviations: LDL, low‐density lipoprotein; NS, non‐significant.

Age and BMI did not differ in the low‐ versus high‐cholesterol absorber subgroups (Table 1). The intakes of dietary cholesterol and fat and the concentrations of serum and lipoprotein lipids were similar in the two subgroups.

### Cholesterol metabolism

In the whole study population, the mean cholesterol absorption efficiency was 35.7% (range 11.9%–63.5%), and it was 1.75‐fold higher in the high‐ versus low‐cholesterol absorbers (*p* < 0.001) (Table 2). In contrast, cholesterol synthesis and faecal neutral sterol levels were higher in the low‐ versus high‐cholesterol absorbers (*p* = 0.002 and *p* = 0.004). The total amount of cholesterol eliminated daily into faeces as faecal neutral sterols and bile acids was also higher in the low‐ versus high‐cholesterol absorbers (*p* = 0.003).

Of the serum biomarkers of cholesterol synthesis, only the serum zymostenol:C ratio was significantly higher in the low‐ versus high‐cholesterol absorbers (*p* = 0.005) (Table 2). The serum biomarkers of cholesterol absorption efficiency were higher in the high‐ versus low‐cholesterol absorbers, as expected.

### Bile acid metabolism

In the whole study population, the mean level of faecal bile acids was 447 ± 274 mg/day, ranging from 159 to 1226 mg/day (Table 2). The primary bile acids (cholic and chenodeoxycholic acids) composed the smallest bile acid group, whereas the secondary bile acids (deoxycholic and lithocholic acids) and the rest of the bile acids (miscellaneous bile acids) composed the two largest groups.

In the low‐ and high‐cholesterol absorber subgroups, faecal bile acid levels were higher in the low (557 ± 324) versus the high absorbers (330 ± 108 mg/day) (*p* = 0.015), ranging from 251 to 1226 mg/day in the low absorbers versus 159–555 mg/day in the high absorbers (Table 2). Levels of the individual primary and secondary bile acids were also significantly higher in the low‐ versus high‐cholesterol absorbers, but their percentage distribution did not differ, suggesting that there was no preferential difference in the elimination of primary or secondary bile acids. Levels of the miscellaneous bile acids did not differ between the low‐ versus high‐cholesterol absorber subgroups.

### Correlations

In the whole study population and in the low‐ and high‐cholesterol absorbers separately, LDL‐C concentrations were similar in the low‐ and high‐cholesterol absorbers and were not associated with the variables of cholesterol metabolism (Table 3).

In the whole study population, cholesterol absorption efficiency correlated negatively with faecal neutral sterol levels (*r* = −0.732, *p* < 0.001) (Fig. 1, upper panel), with cholesterol synthesis (*r* = −0.742, *p* < 0.001) (Fig. 1, middle panel), and with faecal bile acid levels (*r* = −0.547, *p* < 0.001) (Fig. 1, lower panel). These negative correlations were also significant separately in the low‐ and high‐cholesterol absorbers, with the exception of faecal bile acid levels in the low absorbers (Fig. 1, lower panel). Moreover, in the whole study population, levels of faecal bile acids were positively correlated with those of faecal neutral sterols (*r* = 0.654, *p* < 0.001) and with cholesterol synthesis (*r* = 0.850, *p* < 0.001). Faecal bile acid levels were positively correlated with those of cholesterol synthesis also in low (*r* = 0.855, *p* < 0.001) and high (0.715, *p* < 0.01) cholesterol absorbers.

The serum biomarkers of cholesterol absorption efficiency and cholesterol synthesis were negatively correlated (for example, the correlation between serum lathosterol:C and cholestanol:C was *r* = −0.628, *p* < 0.001). Serum lathosterol:C also correlated with the absolute measurement of cholesterol synthesis (*r* = 0.391, *p* < 0.05), and serum cholestanol:C correlated with cholesterol absorption efficiency (*r* = 0.715, *p* < 0.001). Serum zymostenol:C and lathosterol:C ratios correlated significantly with each other in the high cholesterol absorbers (*r* = 0.663, *p* < 0.01).

## Discussion

The main findings of this study were, first, that faecal elimination of neutral sterols and bile acids was reduced in the high cholesterol absorbers, indicating poor/reduced elimination of cholesterol and cholesterol‐derived compounds from the body, potentially increasing the risk of ASCVD [4, 8, 16, 17]. In fact, a relatively long (∼20‐year) prospective follow‐up study showed that decreased faecal bile acid elimination was associated with an increased independent risk of peripheral vascular and carotid artery disease [17]. In addition, low bile acid synthesis characterized men with familial hypercholesterolaemia and CAD compared with those with no CAD [18]. Thus, both studies indicated that impaired bile acid synthesis can be considered atherogenic [17, 18].

Faecal elimination of total cholesterol, calculated as the sum of faecal neutral sterols and bile acids, was on average 595 mg/day higher in the low‐ versus high‐cholesterol absorbers. This was reflected in increased cholesterol synthesis (an average of 548 mg/day more) in the low‐ versus high‐cholesterol absorbers.

Second, bile acid synthesis was dependent on the profile of cholesterol absorption efficiency. It correlated negatively with cholesterol absorption efficiency, and it was significantly higher in the low‐ versus high‐cholesterol absorber subgroup. Bile acid synthesis also correlated positively with cholesterol synthesis, reflecting the interaction between the enzymes HMG CoA‐R and CYP7A1 [21, 25].

Thus, the synthesis of bile acids and cholesterol and faecal neutral sterol elimination correlated with each other. They also correlated negatively with cholesterol absorption efficiency, so that high cholesterol absorption efficiency was associated with low synthesis of bile acids and cholesterol and low elimination of faecal neutral sterols. At the cellular level, it is suggested that in high cholesterol absorbers, the increased load of absorbed chylomicron remnants transports extra cholesterol to the hepatocytes, followed by downregulation of the activities of the hepatic LDL‐receptors and the enzymes HMG CoA‐R and CYP7A1 [41]. As a result, the uptake of LDL‐C to the liver and de novo cholesterol and bile acid synthesis in the hepatocytes are diminished. Also, the reverse transport of cholesterol from the body to the faeces via bile is diminished, of which the elimination of endogenous cholesterol and its metabolites represents the largest proportion (∼72%) [11]. Accordingly, the atherogenic potential of high cholesterol absorption seems to be related to reduced reverse biliary cholesterol transport from the body to faeces and lowered cholesterol and bile acid synthesis.

Third, serum cholesterol and LDL‐C concentrations did not differ in the low‐ versus high‐cholesterol absorber subgroups, a finding also observed earlier (Table 1) [3, 4, 5, 8]. Moreover, there was no correlation between LDL‐C concentrations and the variables of cholesterol metabolism in either the whole study population or separately in the low‐ and high‐cholesterol absorbers (Table 3). These challenging findings probably result from downregulated LDL‐receptor activities and reduced cholesterol synthesis in the high‐ versus low‐absorbers [41], but the exact cellular mechanisms need to be clarified.

Regarding its potential atherogenicity, high cholesterol absorption efficiency should be diagnosed as early as possible in order to induce dietary and lifestyle changes and start adequate cholesterol absorption‐lowering medication with ezetimibe combined with statin when needed. Ezetimibe decreases cholesterol absorption efficiency and increases the reverse cholesterol transport and the elimination of cholesterol as faecal neutral sterols [15, 42]. Phytosterols also inhibit cholesterol absorption, and when combined with ezetimibe, they significantly increase the positive effects of ezetimibe on cholesterol metabolism and lower LDL‐C concentrations [43]. Phytosterol supplementation may therefore be a good solution for high cholesterol absorbers not yet qualifying for cholesterol‐lowering drugs on the basis of their individual risk assessments.

The similar LDL‐C concentrations in low‐ versus high‐cholesterol absorbers give no indication of the presence of high cholesterol absorption efficiency in an individual. However, this can be indicated by a positive family history of CAD and by analysis of the serum biomarkers of cholesterol absorption efficiency and cholesterol synthesis, or the risk alleles of the intestinal ABCG5 and ABCG8 transporters [44]. In general, people with a positive family history of CAD should adhere to healthy dietary and lifestyle habits from early childhood [45].

The study population was in a metabolic steady state according to the definitions [12, 38]. Cholesterol absorption efficiency and cholesterol synthesis, as well as their respective serum biomarkers, correlated negatively with each other, demonstrating that the homeostasis of cholesterol metabolism was also intact [12, 38]. In addition, the serum biomarkers correlated with the absolute metabolic measurements, so that the results obtained can be considered valid.

Although the serum biomarkers of cholesterol synthesis correlated with each other, only the zymostenol:C ratio was lower in the high‐ versus low‐cholesterol absorbers, in the same way as cholesterol synthesis. Since the biomarkers of cholesterol absorption and synthesis have their individual metabolic pathways, it is recommendable to use more than one biomarker to ensure a correct result.

### Limitations and strengths of the study

The size of the study population was restricted because the complicated methods and analyses of the absolute measures of whole‐body cholesterol metabolism limit in general the sizes of the study populations. However, according to the sample‐size calculation, the size of the population was appropriate.

The distribution of men and women in the low‐ and high‐cholesterol absorber subgroups was uneven, but the main baseline demographic, BMI and dietary characteristics and serum and lipoprotein lipid concentrations were similar in both groups. The metabolism of cholesterol followed the low‐high pattern of cholesterol absorption efficiency rather than a gender effect (Tables 1 and 2). According to the earlier studies [4, 5], gender, BMI, serum and lipoprotein lipids or blood glucose concentration did not correlate with cholesterol metabolism, suggesting that the differences in gender or diabetes between the study populations did not interfere with the outcomes of cholesterol metabolism. However, the presence of CAD independently altered cholesterol metabolism in women and men, decreasing cholesterol synthesis and faecal elimination of total sterols of cholesterol origin, that is, neutral sterols and bile acids [4], and increasing cholesterol absorption efficiency [5]. Thus, the differences in gender between the study populations obviously did not interfere with the outcomes of cholesterol metabolism between the low‐ and high‐cholesterol absorption subgroups.

The study data can also be regarded as ‘old’, but the methods used are of ‘gold standard’ and still valid. No recent data exist to execute similar evaluations. The strengths of the study were that both the absolute and relative variables of cholesterol metabolism were analysed, and these ensured the validity of the results. The study population was in a metabolic steady state, and the homeostasis of cholesterol metabolism was intact. In addition, the absolute variables of cholesterol and bile acid metabolism revealed unique and fundamental information, which cannot be obtained by other methods and which is important to implement in patient care.

## Conclusions

The aim of this study was to explore whether different profiles of cholesterol absorption efficiency, that is, low‐ versus high‐cholesterol absorption efficiency, interfere with bile acid synthesis. It turned out that high cholesterol absorption efficiency was associated not only with low synthesis of bile acids but also that of cholesterol and their reduced biliary elimination as faecal neutral sterols and bile acids. In high cholesterol absorbers, poor elimination of cholesterol from the body may indicate an increased risk of atherosclerosis. Thus, early diagnosis of high cholesterol absorption is desirable to enable reduced cholesterol absorption and increased cholesterol elimination by dietary means and ezetimibe treatment, combined with statins when needed.

## Author contributions

**Piia Simonen**: Conceptualization and design of the study; methodology; analysis and interpretation of data; writing—original draft; writing—review and editing. **Ingmar Wester**: Conceptualization and design of the study; methodology; analysis and interpretation of data; visualization; writing—review and editing. **Jyri Lommi**: Conceptualization; acquisition of resources; methodology; writing—review and editing. **Juha Sinisalo**: Conceptualization; acquisition of resources and funding; methodology; writing—review and editing. **Helena Gylling**: Conceptualization and design of the study; methodology; analysis and interpretation of data; writing—original draft; writing—review and editing, visualization.

## Funding information

State Research Funding for Helsinki University Hospital (Y242SK2424)

## Consent

Written informed consent was obtained from each participant.

## Conflict of interest statement

The authors declare no conflicts of interest.

## Ethics statement

The study protocol conformed to the ethics guidelines of the Declaration of Helsinki. The original study protocols were approved at the time by the Ethics Committee of the Second Department of Medicine, University of Helsinki on research on humans (Ethics Committee minutes 11/1991, 17/1994).Trial registrations were not available, because the original studies were performed in 1991–1996, before registration was required in Finland.

## Supporting information




**Table S1**: Detailed characteristics of the two original study populations.

## Data Availability

Data described in the manuscript will be available upon request from the corresponding author.
