# Supplementary material for: High cholesterol absorption efficiency interferes with bile acid metabolism and cholesterol elimination from the body
Source: J Intern Med. 2025 Oct 20;299(5):628–38. doi: 10.1111/joim.70031 (PMC13061100; doi:10.1111/joim.70031)
Supplement: Supplementary file 1 — Table S1: Detailed characteristics of the two original study populations. [file JOIM-299-628-s001.docx]

**JIM-25-0472-R1-Supplemental Table.**

**Supplemental Table.** *Detailed characteristics of the two original study populations.*

| Variables | Men with type 2  diabetes mellitus  n=11^1^ | Women with stable coronary artery disease n=22^2^ | *p*-values |
| --- | --- | --- | --- |
| Age, years | 57.8 ± 1.9 | 51.0 ± 1 | 0.457 |
| Weight, kg | 81.2 ± 3.1 | 68.0 ± 2 | 0.001 |
| Body mass index, kg/m^2^ | 26.5 ± 0.7 | 26.0 ± 0.7 | 0.361 |
|  |  |  |  |
| Serum and lipoprotein lipids, mmol/L | |  |  |
| Serum cholesterol | 5.98 ± 0.22 | 6.01 ± 0.22 | 0.398 |
| VLDL cholesterol  LDL cholesterol | 0.72 ± 0.07  3.83 ± 0.16 | 0.34 ± 0.06  3.66 ± 0.19 | 0.001  0.234 |
| HDL cholesterol | 1.13 ± 0.03 | 1.26 ± 0.06 | 0.096 |
| Serum triglycerides | 2.14 ± 0.17 | 1.40 ± 0.16 | <0.001 |
|  |  |  |  |
| Serum biomarkers of cholesterol synthesis^3^ | |  |  |
| Zymostenol:C | 32.4 ± 2.3 | 14.6 ± 1.5 | <0.001 |
| Lathosterol:C | 185 ± 12.5 | 170 ± 11.6 | 0.043 |
|  |  |  |  |
| Serum biomarkers of cholesterol absorption efficiency^3^ | |  |  |
| Cholestanol:C | 94.4 ± 6.6 | 127 ± 9.2 | 0.003 |
| Sitosterol:C | 110 ± 12.2 | 147 ± 12.7 | 0.033 |
|  |  |  |  |
| Cholesterol and bile acid metabolism | |  |  |
| Cholesterol absorption efficiency, % | 25.3 ± 2.1 | 42.2 ± 2.1 | <0.001 |
| Cholesterol synthesis, mg/kg/day | 18.2 ± 1.6 | 11.6 ± 0.81 | 0.001 |
| Faecal neutral sterols, mg/kg/day | 13.5 ± 1.1 | 9.80 ± 0.68 | 0.002 |
| Faecal bile acids, mg/kg/day | 8.6 ± 1.1 | 4.88 ± 0.34 | 0.004 |
|  |  |  |  |

Mean ± standard error of mean. ^1^Ref. 27, ^2^Ref. 28. ^3^10^2^ µmol/mmol of cholesterol. Abbreviations: C = cholesterol, HDL = high-density lipoprotein, LDL = low-density lipoprotein, VLDL = very low density lipoprotein.
